# Isobutyryl-coenzyme a dehydrogenase deficiency: disease, or non-disease?

**DOI:** 10.1186/s13023-026-04207-7

**Published:** 2026-01-29

**Authors:** María Daniela Santacruz Reyes, Jörn Oliver Sass

**Affiliations:** Department of Natural Sciences & Institute for Functional Gene Analytics (IFGA), Research Group Inborn Errors of Metabolism, Bonn-Rhein Sieg University of Applied Sciences, Von-Liebig-Str. 20, 53359 Rheinbach, Germany

**Keywords:** Isobutyryl-CoA dehydrogenase deficiency, Isobutyrylglycinuria, *ACAD8*, Valine metabolism, Newborn screening, Acylcarnitine, Clinical phenotype, Hepatic steatosis

## Abstract

**Background:**

Isobutyryl-coenzyme A dehydrogenase deficiency (IBDD) is a rare inborn error of valine metabolism caused by variants in the *ACAD8* gene. Since its initial description in 1998, a wide range of clinical features has been reported, but the disease status and clinical significance of IBDD remain under debate. We systematically studied all published cases of IBDD to provide an overview of the reported phenotype and molecular spectrum.

**Results:**

A comprehensive literature review identified 172 individuals with IBDD reported up to December 2024. Seven children were diagnosed following selective screening due to family history or clinical suspicion, while 165 were identified through expanded newborn screening programs. Elevated blood or plasma C4-acylcarnitine was observed universally, and isobutyrylglycinuria was a common but not invariable urinary marker. Of these 172 individuals, 146 were asymptomatic at follow-up, whereas 26 presented with diverse, non-specific manifestations, including motor delay, failure to thrive, muscular hypotonia, speech delay, developmental delay, and anemia—the latter being the most frequently reported abnormality. Biallelic pathogenic variants in *ACAD8* were identified in most cases with available genetic information, with c.286G > A p.(Gly96Ser) emerging as the most prevalent variant, predominantly among individuals of Chinese origin. Notably, altered biochemical markers of liver function were reported in 19 individuals, including 18 with isolated elevations of serum transaminases and γ-glutamyl transferase. One 11-year-old boy exhibited hepatomegaly and ultrasound findings suggestive of hepatic steatosis, along with markedly elevated transaminase levels. Hepatic steatosis has also been observed in an IBDD mouse model, suggesting a potential link between IBDD and liver involvement.

**Conclusions:**

Most individuals with IBDD remain asymptomatic following detection through newborn screening, yet a minority develop heterogeneous clinical features. Our overview highlights that some liver enzyme abnormalities and hepatic steatosis may occur in some individuals with IBDD. These findings suggest that further research is warranted to clarify possible hepatic implications of IBDD and to determine whether long-term monitoring of affected individuals should be considered, particularly in light of ongoing discussions about the appropriateness of IBDD as a target condition in newborn screening programs.

**Supplementary Information:**

The online version contains supplementary material available at 10.1186/s13023-026-04207-7.

## Introduction

Inborn errors of metabolism encompass genetic disorders that are typically characterized by impaired metabolic pathways. Mostly, these deficiencies lead to the accumulation of toxic substrates or intermediate metabolites, often resulting in non-specific or complex clinical manifestations [1].

**Fig. 1:**
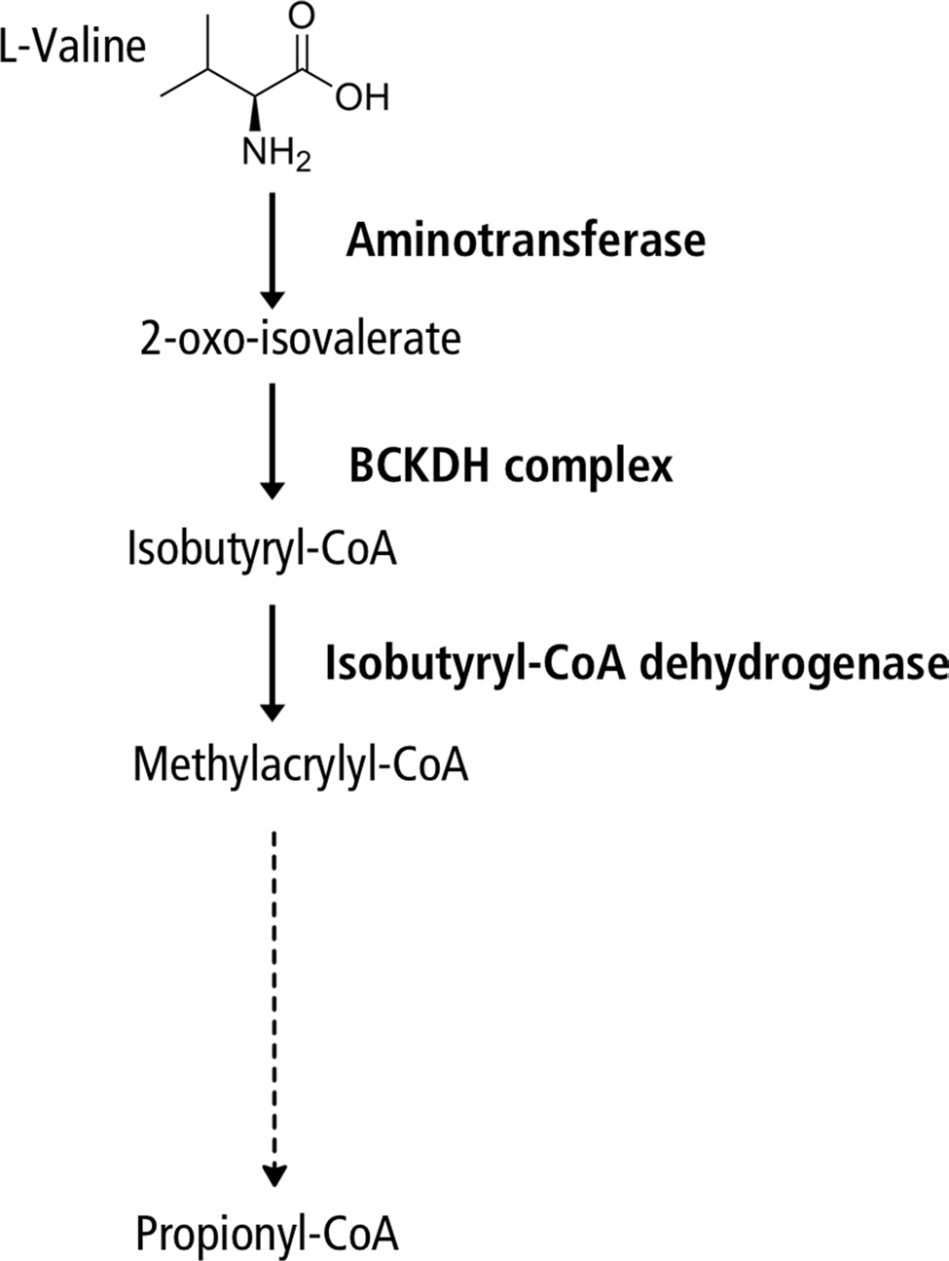
Valine catabolism pathway. Schematic representation of the metabolic pathway of valine degradation

Human isobutyryl-coenzyme A (CoA) dehydrogenase deficiency (IBDD) is a rare autosomal recessive disorder belonging to this group. It is caused by biallelic variants in the *ACAD8* gene, located on chromosome 11q25. This gene consists of 11 exons and encodes isobutyryl-CoA dehydrogenase (IBD), a mitochondrial enzyme composed of 415 amino acids. The enzyme catalyzes the conversion of isobutyryl-CoA (isobutyryl-CoA) to methacrylyl-CoA, a key step in the catabolism of the branched-chain amino acid valine, ultimately linking to the tricarboxylic acid cycle (Fig. 1). Not only variations of the enzyme that affect substrate binding may decrease or abolish IBD enzyme activity, but also those that disrupt protein folding and/or stability [2].

IBDD was first described in 1998 in a 2-year-old female who developed anemia, dilated cardiomyopathy, and carnitine deficiency after a period of normal development during her first year of life [3]. Following treatment, she became carnitine-dependent but exhibited normal growth and development. According to a later follow-up referenced by Oglesbee et al. [4], the patient remained carnitine-dependent and clinically stable up to around age 11 (personal communication, S. Cederbaum, 2006, cited in reference [4]).

Diagnosis of IBDD has mostly been accomplished *via* newborn screening (NBS), as tandem mass spectrometry enables the detection of elevated C4 acylcarnitine, a key biochemical marker suggestive of the disorder [5]. However, the inclusion of C4 acylcarnitines in screening panels is inconsistent, and the classification of IBDD varies across international NBS programs, where it is often not included or listed as a secondary target [6]. While additional findings—such as carnitine deficiency and increased urinary isobutyrylglycine—can support diagnosis, these markers are inconsistently reported [7, 4]. Confirmatory diagnosis typically relies on genetic testing.

Although many diagnosed individuals have shown normal development, a subset of reported cases presented with clinical symptoms of varying severity, including developmental delay and metabolic disturbances [8]. However, the extent to which these manifestations are directly attributable to IBDD remains uncertain, and the natural history of the disorder is still poorly understood. This uncertainty highlights a need to systematically evaluate the available evidence regarding the phenotype of individuals with IBDD.

A “disease” is defined as a pathological condition requiring medical attention, characterized by identifiable abnormalities that underlie clinical symptoms. In contrast, a “disorder” is a broader term that comprises also a combination of symptoms without known clinical consequences [9, 10]. Given the predominantly asymptomatic nature of IBDD and the lack of consistent clinical manifestations, there remains ongoing debate about whether IBDD constitutes a disease or is merely a biochemical disorder without significant clinical impact [11, 12].

Therefore, a systematic assessment of patient reports was needed to better understand the clinical relevance of IBDD. This study aims to explore the clinical relevance of IBDD, addressing key questions regarding its classification as a disease versus a (merely biochemical) disorder, the impact of NBS availability on detection rates, and the pathogenicity of the most commonly identified *ACAD8* variants. By analyzing available literature, this work seeks to clarify whether IBDD represents a clinically significant condition or remains a biochemical finding of uncertain significance.

## Methods

### Literature search and study selection

A systematic literature search in PubMed was performed using the terms “isobutyryl-CoA dehydrogenase”, “*ACAD8*”, and “isobutyrylglycinuria”, aiming for information on the clinical courses and genetic characteristics of all individuals with isobutyryl-CoA dehydrogenase deficiency reported in the literature. The search was performed from October 2024 on and was complemented by searches in the Human Gene Mutation Database (HGMD^®^) http://www.hgmd.cf.ac.uk/. The most recent search was performed in November 2024. Patient data—including clinical features and genetic variants of the *ACAD8* gene—were extracted from the identified publications and recorded in a standardized data collection template. The following data items were collected when available: age at diagnosis, sex, family history, biochemical markers, clinical manifestations, genetic variants, treatment, and outcomes. All patients with metabolically, enzymatically, and/or genetically confirmed isobutyryl-CoA dehydrogenase deficiency on whom sufficient clinical or genetic information was found, were included in this study. In addition, studies that identified individuals with IBDD only through reporting case counts, without individual clinical description, were also included to estimate the approximate number of patients reported in the literature. Studies were excluded only if IBDD was not mentioned in the publication. The list of publications used specifically for patient data extraction is provided as Annex 1.

**Fig. 2:**
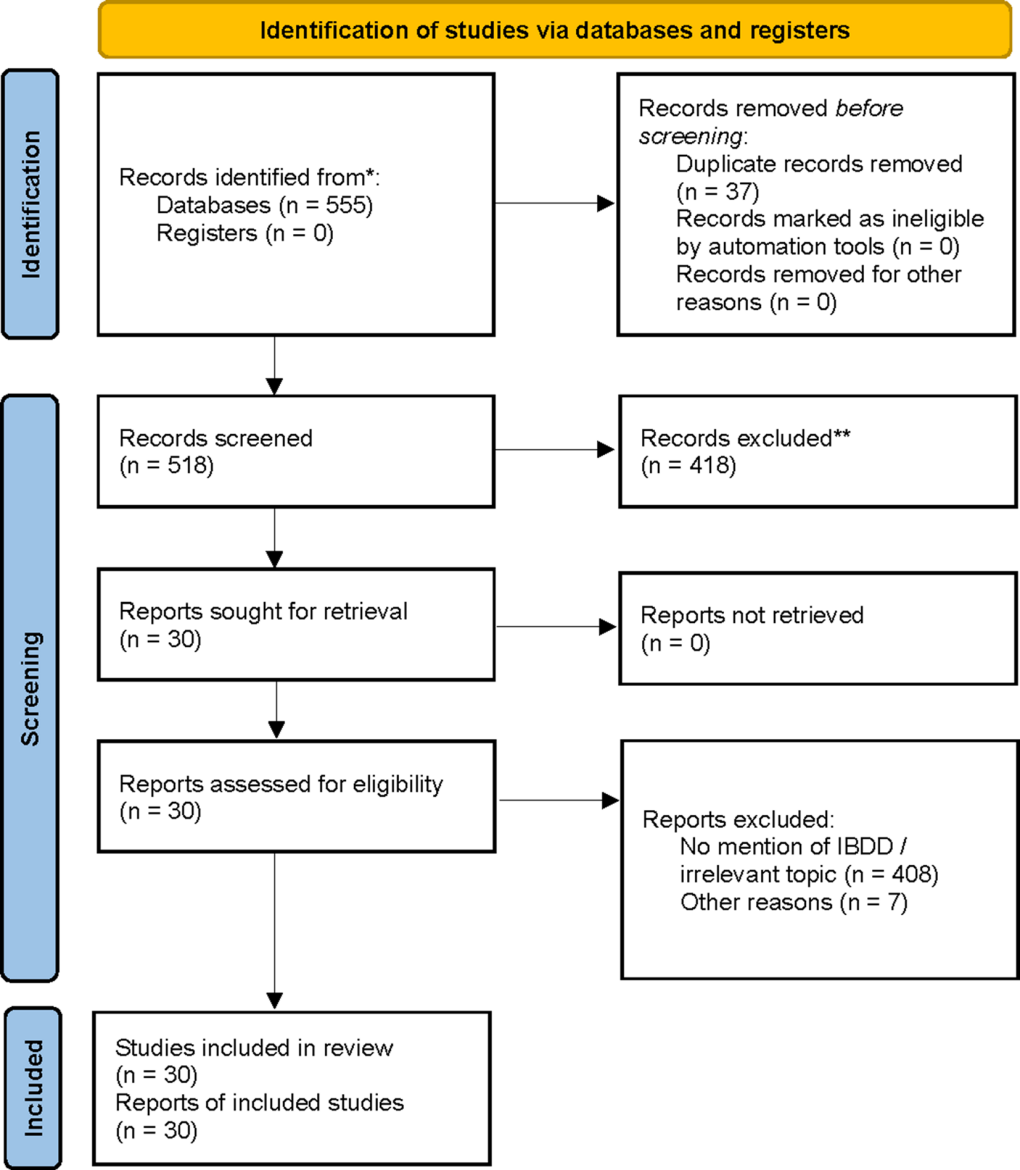
PRISMA 2020 flow diagram for new systematic reviews which included searches of databases and registers only. Flow diagram of the study selection process for the literature review on isobutyryl-CoA dehydrogenase deficiency (IBDD) following PRISMA 2020 guidelines [33]. Numbers indicate records identified, screened, excluded, and included at each stage

The study selection process, including records identified, screened, and included, is summarized in the PRISMA 2020 flow diagram (Fig. 2).

### Variant pathogenicity assessment

The pathogenic potential of the identified *ACAD8* variants was assessed using multiple in silico prediction tools. The tools employed included Mutation Taster (http://www.mutationtaster.org/), SIFT (https://sift.bii.a-star.edu.sg/), PolyPhen-2 (http://genetics.bwh.harvard.edu/pph2/), GeneBe (http://www.gene-be.com/), Franklin (https://franklin.genoox.com/), and Provean (accessed via GeneBe; http://provean.jcvi.org/).

### Secondary literature search

To ensure comprehensive coverage of the literature, an additional search was performed in November, 2024, using the terms “extended newborn screening” and “inborn error of metabolism”. Publications identified through this secondary search were subsequently examined manually for mentions of isobutyryl-CoA dehydrogenase, *ACAD8*, and isobutyrylglycinuria.

### Statistical analysis

To compare the frequency of clinical symptoms between patient groups, we performed statistical analysis using Fisher’s Exact Test [13]. This method is suitable for small sample sizes and categorical variables, making it appropriate for analyzing the clinical data collected in this study. Narrative synthesis was performed for descriptive clinical and genetic data.

### Population group classification

To facilitate the analysis and comparison of genetic data, the patient population was grouped according to the population categories defined by the Genome Aggregation Data-base (gnomAD; https://gnomad.broadinstitute.org/). This classification allowed for a standardized categorization of populations across the different studies reviewed, despite variations in how populations were originally reported in the literature. Based on this framework, populations were organized into the following regional groups: East Asia (in-cluding Chinese and Korean individuals), South Asia (Pakistani and Indian), Middle East & North Africa (MENA) (including Turkish, Middle Eastern, and North African populations), Europe (further subdivided into Northern Europe for Danish and North-West for German individuals, and Central/Western Europe for general European and Caucasian entries), Hispanic/Latin America, Indigenous America (Native American), and Sub-Saharan Africa (Black individuals). This regional approach was chosen to reflect geographic origin and genetic proximity, ensuring a consistent and interpretable analysis of variant distribution across populations.

### Risk of bias assessment

Case reports and case series constituted the primary data source for this review; therefore, no standardized risk of bias tool could be uniformly applied. Risk of bias was qualitatively considered based on completeness of reporting.

## Results

### Sex distribution

Information regarding sex was given for 55 of the studied 172 individuals. Of those, 31 were female (18.1%) and 24 were male (14.0%). In the remaining 117 cases (68.4%), sex was not specified. This substantial proportion of missing data limits any meaningful interpretation of sex-related trends in IBDD.

### Populations

Population background was reported inconsistently across the examined reports. To facilitate comparison, the available ancestry information was reclassified according to the gnomAD population groups. The largest group identified was East Asian, comprising 92 patients (51.7%), of which 88 were Chinese and 4 were Korean. Other populations included European (9 cases, 5.06%), Hispanic (8 cases, 4.49%), African (5 cases, 2.78%), Middle Eastern (4 cases, 2.25%), and Native American (2 cases, 1.12%). *Population information was not reported for the remaining 52 individuals.*

### Diagnostic approach

Across the reviewed studies, a total of 172 individuals with IBDD was identified. Most of the patients (165) were diagnosed through NBS, highlighting the central role of metabolic screening in early detection. Seven patients were diagnosed *via* selective laboratory screening processes, initiated by clinical findings or family history.

### Patient condition

**Fig. 3:**
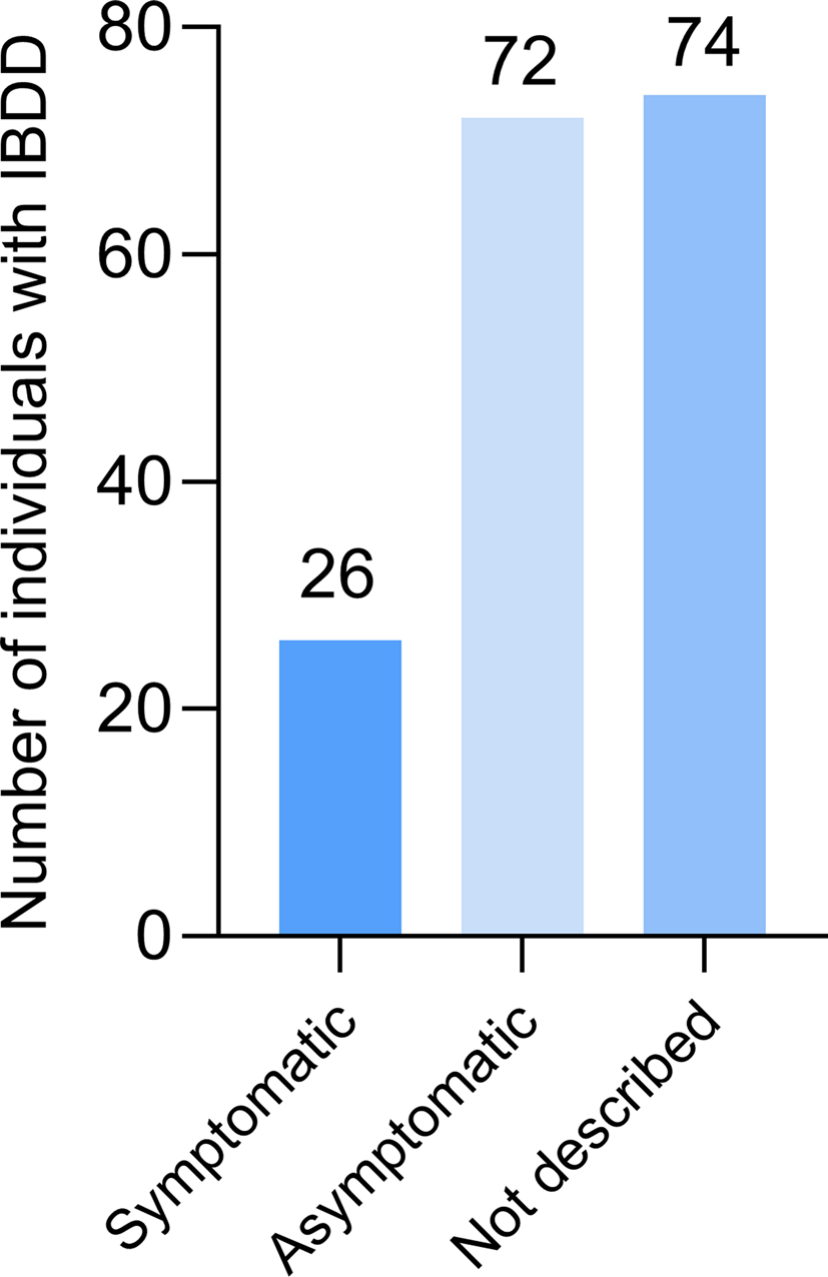
Clinical status of individuals diagnosed with IBDD. Overview of the reported clinical presentation among 172 individuals with isobutyryl-CoA dehydrogenase deficiency (IBDD). Of these, 26 individuals (15.1%) were described as symptomatic, 72 (41.9%) were asymptomatic, and 74 (43.0%) lacked clinical detail in the literature, having been mentioned as diagnosed through newborn screening (NBS) without further characterization

Among the 172 individuals of the study cohort, 26 were reported as symptomatic, 72 remained asymptomatic, and for 74 individuals no clinical information was available. This distribution suggests that symptomatic presentation is relatively infrequent. However, the wide range of symptoms observed among the affected individuals may also reflect possible roles of IBDD as a modifying factor (Fig. 3).

### Clinical findings

**Fig. 4:**
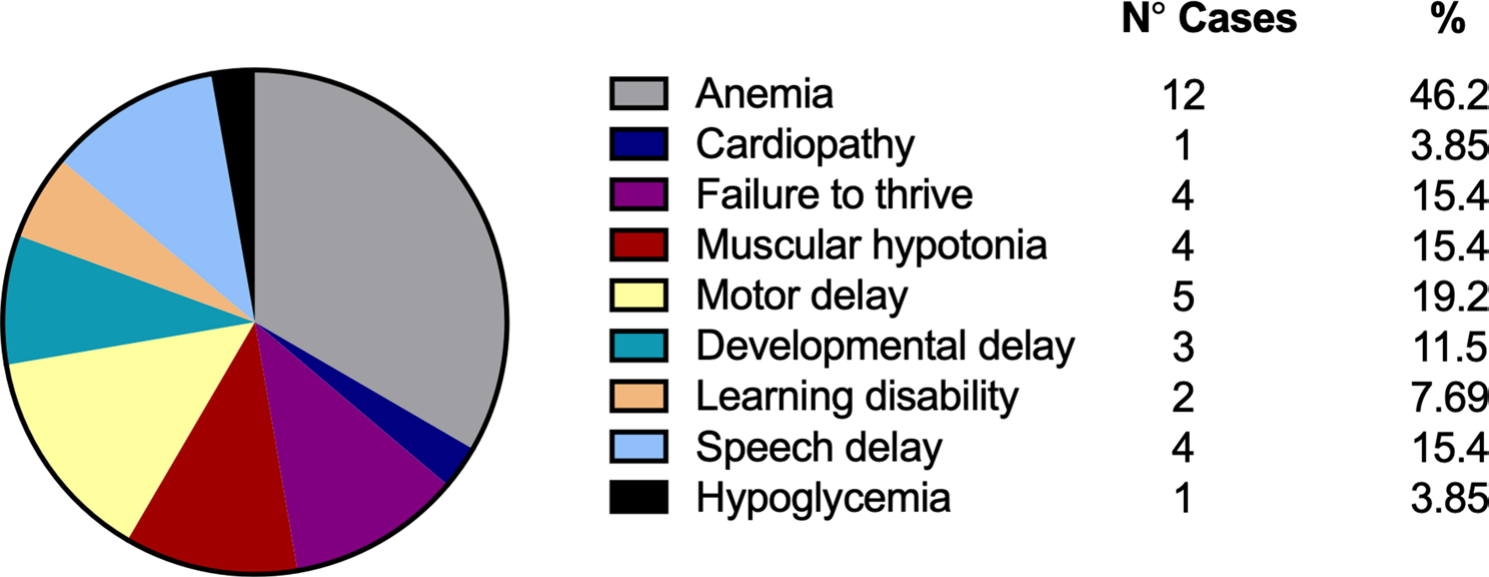
Distribution of clinical features among symptomatic IBDD patients. Proportional representation of the various clinical manifestations observed in the 26 symptomatic individuals with isobutyryl-CoA dehydrogenase deficiency (IBDD). The figure illustrates the heterogeneity of presentations among this subset

Within the symptomatic group of 26 individuals, a variety of clinical features was observed. The most common finding was anemia, reported in 12 patients (46.2%). Other manifestations included motor delay (5 patients, 19.2%), failure to thrive (4 patients, 15.4%), muscular hypotonia (4 patients, 15.4%), and speech delay (4 patients, 15.4%). Additionally, developmental delay was seen in 3 patients (11.5%) and learning disabilities in 2 patients (7.69%). Less common features included cardiopathy (1 patient, 3.85%) and hypoglycemia (1 patient, 3.85%). These findings illustrate the heterogeneity of IBDD manifestations and the broad clinical spectrum of the disorder (Fig. 4).

### Symptom distribution

Clinical findings were reported in both NBS-diagnosed patients (21 individuals) and in patients identified by selective screening (5 individuals). Of the 7 selectively diagnosed patients, 5 were symptomatic, while 2 remained asymptomatic and were tested because of their family history.

Anemia was the most common symptom in both groups, found in 47.2% of NBS-diagnosed symptomatic individuals and 40.0% of those diagnosed *via* selective screening. Other shared symptoms included failure to thrive (14.3% in the NBS group vs. 20.0% in the selective screening group) and muscular hypotonia (14.3% vs. 20.0%, respectively).

Developmental delay was observed in both groups, affecting 9.50% of NBS-diagnosed symptomatic individuals and 20.0% of those diagnosed *via* selective screening. Motor delay, however, was reported only among NBS-identified patients (19.1%). In contrast, cardiopathy and hypoglycemia were exclusive to the selective screening group, each occurring in 20.0% of these individuals and not reported among those identified through NBS.

Additionally, speech delay (9.52% NBS vs. 40.0% selective screening) and learning disabilities (none in NBS vs. 40.0% selective screening) were more prevalent among the individuals diagnosed *via* selective screening.

### Statistical analysis

Clinical symptom frequencies were compared between individuals diagnosed through newborn screening (NBS) and those identified by selective screening. Although some symptoms appeared to differ in frequency between the groups, none of these differences reached statistical significance using Fisher’s Exact Test (all *p* > 0.05), likely due to the small overall sample size, particularly within the selectively diagnosed group (*n* = 5).

Anemia was the most frequent symptom overall, present in 47.6% of NBS-diagnosed individuals and 40.0% of selectively diagnosed patients (*p* = 1.0000). Motor delay was observed exclusively in the NBS group (23.8%), while cardiopathy and hypoglycemia were only reported in selectively diagnosed individuals (20.0% each); however, these differences did not reach statistical significance (*p* = 0.5451 and *p* = 0.1923, respectively). Other symptoms, such as muscular hypotonia, failure to thrive, and developmental delay, were present in both groups with comparable frequencies.

The distribution of certain symptoms across groups, even if not statistically significant, may indicate trends that warrant further investigation in larger, more representative cohorts to better understand possible phenotypic differences.

### Age at diagnosis

Age at diagnosis was explicitly reported for 33 patients. Among these, 26 (15.1%) were diagnosed *via* newborn screening (NBS) with a specific age provided (e.g., “3 days” or “5 days”).

Later diagnoses were reported in individuals aged between 12 months and 11 years. These were the cases identified through selective testing prompted by clinical symptoms or familial relation to a known case.

The remaining 139 patients diagnosed via NBS (80.8%) were labeled simply as “newborn,” without a precise age given.

### Age at last follow-up

Follow-up data was available for 42 patients, ranging from 18 days to 11 years. Most cases were followed for less than 3 years: 21 patients (50.0%) had follow-up data ending before the age of 2, while only 6 patients (14.3%) were followed for longer than 6 years.

No follow-up data was available for the remaining 130 patients, limiting a full understanding of the natural history and long-term outcomes of the disorder.

### C4 acylcarnitine analysis

C4 acylcarnitine is the primary biomarker for detecting IBDD during newborn screening. All 172 patients were tested for elevated C4 acylcarnitine levels, which led to the preliminary diagnosis. For 144 patients, an elevated concentration of C4 acylcarnitine was specifically documented; for 26 patients, elevation of C4 acylcarnitine was reported but exact values were not provided.

### Urinary organic acids – Isobutyrylglycine analysis

Isobutyrylglycine is a urinary metabolite associated with IBDD, but it is not consistently elevated in affected individuals, limiting its reliability as a standalone confirmatory test. Among the reviewed cases, 28 patients had elevated isobutyrylglycine levels. In contrast, 33 patients had normal or unremarkable findings, with descriptors including “traces,” “within range,” “indistinctive,” or “unremarkable.” For the remaining 111 patients, the measurement was either not performed or not reported. The latter may also indicate different analytics sensitivities for this biomarker in the various laboratories involved.

### Variants in the *ACAD8* gene

Of the 172 individuals, 101 had reported genetic variant information, while the reports on the remaining 71 did not include specific genetic data—though some studies referenced aggregated findings [14–16]. A total of 64 distinct variants in the *ACAD8* gene was identified. The most frequently reported was c.286G > A, p.(Gly96Ser), found in 44 patients: 12 homozygous, 31 compound heterozygous, and 1 with a single allele identified (the second allele undetected, but the biochemical profile supported the diagnosis of IBDD). Several studies that did not provide individual genotypes also mentioned this variant as frequently observed [1415,16]. It was reported exclusively in Chinese.

Other recurrent variants included c.1000 C > T, p.(Arg334Cys) in 17 patients, identified in individuals from East Asian and European origins; c.1176G > T, p.(Arg392Ser) in eight patients, all from East Asian populations, c.455T > C, p.(Met152Thr) in six patients, reported in individuals from East Asian, European and Native American backgrounds; c.444G > T, p.(Pro148Pro) in five patients, all of East Asian origin; and c.500delG, p.(Ser167Metfs*7) also in 5 patients, reported exclusively in East Asian individuals.

Additional variants were reported in single individuals or small groups, underscoring the extensive genetic heterogeneity associated with *IBDD*.

Among the 64 variants, the majority (45) were missense changes—single nucleotide substitutions that alter the amino acid sequence and may impact protein folding or function. Several variants are expected to affect RNA splicing, including splice donor (e.g., c.1092 + 1G > A, c.705 + 1G > A) and splice acceptor sites (e.g., c.110–2 A > T, c.842-1G > A), as well as those located in splice regions. Six frameshift variants were identified (e.g., c.163-164insCT, c.360delA, c.413delA, c.4_5delCT, c.500delG, c.712delT), which are likely to disrupt the reading frame and to result in truncated proteins. Additionally, two stop-gained variants, two start-loss variants, one in-frame deletion, and one synonymous variant were observed. Some variants had overlapping effects, such as a missense change also impacting splicing, illustrating the complexity of their functional consequences.

**Fig. 5:**
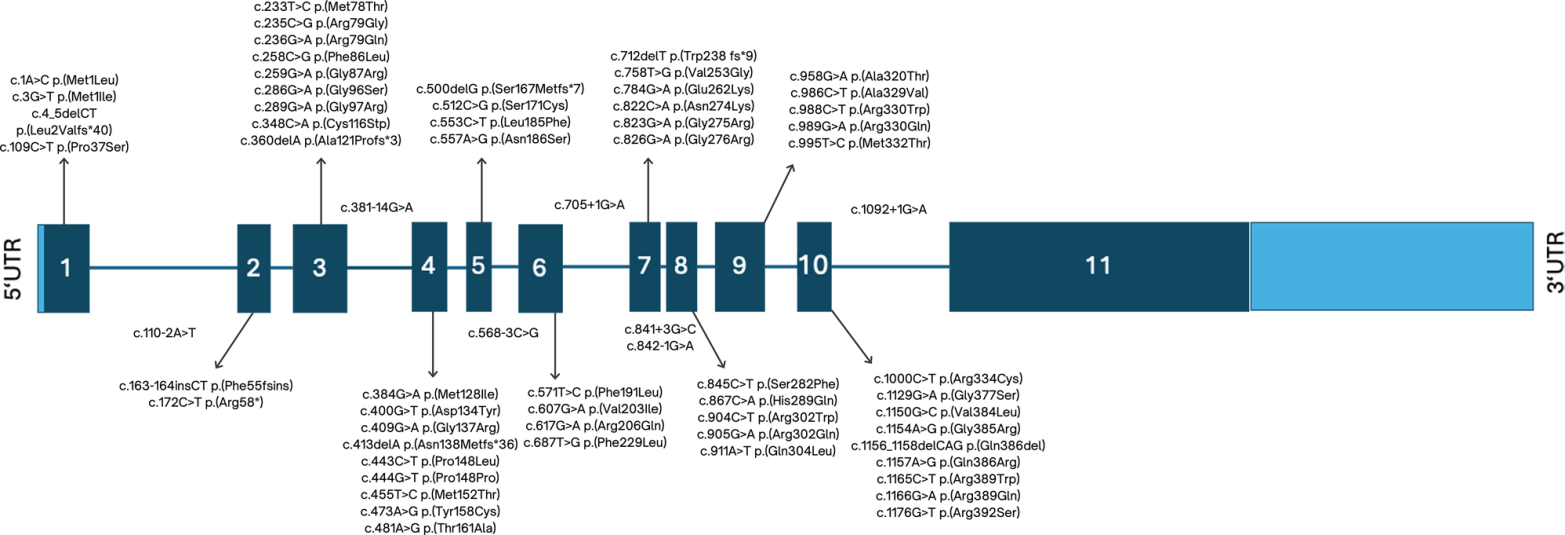
Schematic representation of the *ACAD8* gene and distribution of reported variants Scaled illustration of the *ACAD8* gene, showing all 11 exons and intervening introns. The locations of 64 reported pathogenic or likely pathogenic variants associated with isobutyryl-CoA dehydrogenase deficiency (IBDD) are mapped across the gene

The 64 *ACAD8* variants were distributed across exons 1–10 and several intronic regions. No variant underlying IBDD was reported for exon 11. Exons 3 and 4 harbored the highest number of distinct variants (9 each), followed by exon 10 (8 variants). Exons 1, 2, 5, and 6 each had 4 variants; exon 7 had 6; exon 8 had 5; and exon 9 had 6. Seven intronic variants were also reported (Fig. 5).

## Discussion

To date, to our knowledge, 172 individuals with isobutyryl-CoA dehydrogenase deficiency have been reported across the literature, though many were not clinically characterized in detail. Among those with available clinical information, most remained asymptomatic, particularly those identified through newborn screening. Only 26 individuals (15.1%) were reported to have symptoms, with anemia being the most frequently documented finding. Other manifestations included motor and developmental delays, hypotonia, failure to thrive, speech delay, and learning disabilities. More severe outcomes such as cardiomyopathy and hypoglycemia were reported exclusively in selectively diagnosed individuals [3, 17]. Some case reports also noted nonspecific symptoms such as vomiting, often in the context of infections, which may contribute to metabolic stress in vulnerable patients.

In this overview, anemia emerged as the most frequently reported symptom among symptomatic individuals diagnosed with IBDD. While this finding is notable, there is currently no established mechanistic or clinical link between IBDD and anemia, and the disorder is not known to disrupt hematological pathways. The earliest report of anemia in an IBDD patient was in the index case described by Roe et al. [3], and several subsequent reports—particularly from large Chinese NBS cohorts—also listed anemia among clinical features. However, given the geographic concentration of reported cases in rural China, where micronutrient deficiencies are well-documented, this association may reflect contextual rather than disease-specific factors. A systematic review by Wong et al. [18] reported anemia prevalence rates ranging from 8% to 40% among rural Chinese children, with both iron and vitamin B12 deficiencies significantly more common in lower-income areas. More recent studies have supported this trend: Sheng et al. [19] observed a high prevalence of vitamin B12 deficiency among toddlers in impoverished rural regions, largely attributed to low intake of animal-sourced foods, and Xu et al. [20] identified persistent nutritional burdens among children and adolescents in under-resourced populations. Given this context, the frequency of anemia observed in IBDD patients may reflect broader environmental and nutritional conditions rather than a specific feature of IBDD itself.

Biochemical analysis remains the cornerstone of IBDD detection, particularly within newborn screening protocols. All reviewed cases reported elevated C4 acylcarnitine levels, which serve as the primary biomarker prompting further investigation. However, as noted in several studies (e.g [21, 22]), this elevation is not specific to IBDD and can also be observed in other metabolic disorders such as short-chain acyl-CoA dehydrogenase (SCAD) deficiency, complicating early diagnostic interpretation.

An additional diagnostic challenge lies in the variation of C4 acylcarnitine cutoff values across screening programs, as discussed by Feng et al. [14]. Reported thresholds differed across studies, which may influence detection rates. However, this variability may also reflect true population differences due to factors such as diet, metabolic background, or differences in analytical platforms [23, 24]. Similarly, Oglesbee et al. [4] emphasized that while elevated C4 may raise suspicion, it is insufficient on its own for diagnosis and should always be confirmed through further biochemical and genetic testing.

Urinary isobutyrylglycine, another frequently referenced biomarker, demonstrated inconsistent diagnostic utility. Of the individuals for whom data was available, only a subset showed elevated levels, while others had normal or unremarkable results. This variability, also noted in reports by Knerr et al. [12], Lin et al. [25], and Sadat et al. [5], significantly limits the reliability of isobutyrylglycine as a standalone confirmatory marker. Additionally, many studies did not report whether this metabolite was tested, which may reflect both limited analytical sensitivity and the variability in the presentation. These findings align with strategies applied across multiple studies (e.g [4, 5, 21]). , that a combination of biochemical and molecular approaches—including acylcarnitine profiling, urinary organic acid analysis, and *ACAD8* genetic testing—is essential for accurate and definitive diagnosis of IBDD.

IBDD has been reported as a panethnic condition, though in our overview, over half of all described cases (51.7%) were individuals of East Asian origin, the majority from China. Several large-scale NBS studies have been conducted in China, including those by Feng et al. [14] and Zhuang et al. [15], which have contributed substantially to the literature. In one such cohort, the variant c.286G > A, p.(Gly96Ser), accounted for 44% of alleles and was observed almost exclusively in Chinese patients. While this may be explained by differences in NBS panel composition, diagnostic criteria, and reporting practices, regional clustering may also play a role. It remains unclear whether the apparent predominance of cases in East Asia reflects true prevalence or detection bias. Notably, ancestry information was unavailable for 27.5% of patients in our dataset, limiting conclusions about global distribution. More consistent reporting of demographic data will be essential for improving our understanding of the epidemiology of IBDD.

A critical limitation across the reviewed literature is the lack of consistent and long-term follow-up data for individuals diagnosed with IBDD. Of the 172 patients identified in this overview, only 42 (24.4%) had documented follow-up information, and among those, half were monitored for less than two years. This limited timeframe constrains the ability to draw firm conclusions about the natural history of the disorder, particularly in asymptomatic individuals or those with transient clinical symptoms. Several studies (e.g [5, 23]). , acknowledge this issue, noting that the duration and quality of follow-up are often insufficient to assess whether mild biochemical findings evolve into clinically relevant outcomes over time. Furthermore, the absence of standardized follow-up protocols complicates comparisons across studies and may lead to underreporting of delayed-onset symptoms or emerging comorbidities. Whether IBDD has a natural disease course at all, or whether it represents a benign metabolic variation in most individuals, remains an open question.

This uncertainty also intersects with broader discussions about the goals of newborn screening. NBS is primarily intended to detect conditions that require early intervention in infancy or early childhood. In the case of IBDD, where symptoms—if present—may occur later in life or not at all, the clinical value of early identification is less certain. This has raised concerns about the risk of medicalizing individuals who may remain asymptomatic, especially when no specific treatment is indicated. These factors have contributed to differences in international screening practices. While some countries screen for IBDD and SCADD, others—such as Germany—exclude them from NBS panels, citing insufficient clinical utility and the potential for harm [26]. These variations reflect the ongoing uncertainty surrounding how best to interpret and act on such findings.

While IBDD is typically considered a mild or asymptomatic condition, a subset of patients presents with additional clinical or genetic findings that may suggest a broader metabolic involvement or modulating factors. Hepatic dysfunction has emerged as one such area of interest. In our overview, 18 individuals exhibited altered liver function biomarkers, including elevated ALT and AST, suggestive of hepatic involvement. Notably, one patient—a boy diagnosed at age 11—harbored compound-heterozygous variants in the *ACAD8* gene (c.512 C > G, c.822 C > A), however, along with a heterozygous *ETFDH* gene variant. He presented with hepatomegaly and ultrasound findings consistent with hepatic steatosis [27]. This aligns with findings from a mouse model of IBDD [28], in which hepatic steatosis was also observed, reinforcing a possible metabolic connection between IBDD and liver dysfunction. Given these findings, the use of abdominal ultrasound may be a valuable tool in the clinical evaluation of patients with IBDD. Although a definitive causal relationship between IBDD and liver disease has not been established, emerging evidence from both human and animal studies included in this review suggests a potential link worth further investigation. Additionally, considering the increasing global prevalence of pediatric liver disease, particularly metabolic dysfunction-associated steatotic liver disease (MASLD), even among otherwise healthy children [29], it becomes prudent to include liver assessment in the follow-up of IBDD patients. Abdominal ultrasound offers a non-invasive, cost-effective, pain-free, and widely available method for detecting liver changes such as steatosis or hepatomegaly. Given its safety and diagnostic value, this imaging modality may serve as a reasonable screening tool to identify early hepatic alterations in this patient population.

Several individuals diagnosed with IBDD were reported to have a second, most likely unrelated diagnosis. These ranged from common, mostly non-genetic conditions such as asthma and ketotic hypoglycemia, to confirmed genetic syndromes including chromosome 18 deletion [11], Wiedemann-Steiner syndrome [14], and glutaric aciduria type I [30]. In a few cases, coexisting variants were identified in genes involved in fatty acid oxidation, such as *ACADS* [4] or *ETFDH* [27], suggesting a potential additive metabolic burden. Other findings, including congenital heart abnormalities [31] and autism spectrum disorder [32], were reported in individuals with IBDD but without a clear mechanistic link to the underlying metabolic defect.

Collectively, these atypical presentations and comorbidities might suggest that IBDD, while often benign in isolation, may act as a modifying or contributing factor in the context of other metabolic or neurological conditions. Comprehensive metabolic and genetic evaluations are warranted in such cases to better understand the full clinical spectrum and potential interactions at play. A summary of the coexisting diagnoses observed in the reported cases is presented in Table 1.

**Table 1:** Reported coexisting conditions in individuals with IBDD

Reference number & authors	Second diagnosis	Consanguinity	Characteristic
22 (Pena et al. 2012)	Chromosome 18 deletion	NA	Hereditary
24 (Pena et al. 2012)	Asthma	NA	-
30 (Santa et al. 2017)	Ketotic hypoglycemia		-
32 (Oglesbee et al. 2007)	*ACADS* gene variants	NA	Genetic
40 (Oglesbee et al. 2007)	*ACADS* gene variants	NA	Genetic
57 (Eleftheriadou et al. 2020)	Autism	Yes	-
59 (Pedersen et al. 2006)	Mild branch peripherial pulmonary stenosis	No	-
72 (Popek et al. 2010)	Glutaric aciduria Type 1	Yes	Genetic
73 (Tummolo et al. 2022)	Heterozygous *ETFDH* gene variant	No	Genetic
115 (Feng et al. 2021)	Wiedemann-Steiner syndrome	NA	De novo variant

Summary of additional diagnoses described in individuals with IBDD. Conditions include genetic syndromes, metabolic disorders, and unrelated clinical findings. Reference numbers correspond to individual entries in the reviewed literature. “NA” indicates that consanguinity status was not reported

The *ACAD8* gene, encoding isobutyryl-CoA dehydrogenase, exhibits notable genetic heterogeneity in reported IBDD cases, with 64 distinct variants identified, including missense, frameshift, splice site, nonsense, and start-loss mutations. The missense variant c.286G > A p.(Gly96Ser) is the most frequently reported, predominantly in Chinese cohorts, suggesting a potential founder effect [14, 15].

Establishing a robust genotype-phenotype correlation remains challenging. Most patients, including those with predicted deleterious variants, remained asymptomatic, pointing to potential compensatory metabolic mechanisms or gene-environment interactions [12, 11]. However, in rare cases with clinical symptoms, such as those with hepatic involvement or developmental delays, compound heterozygosity or co-occurring genetic variants (e.g., in *ETFDH*) may modulate the clinical presentation [27]. These observations emphasize the importance of combining biochemical, genetic, and clinical data to understand the spectrum of IBDD and highlight the need for future functional studies to evaluate the precise impact of individual *ACAD8* variants.

Newborn screening has played a fundamental role in the identification of IBDD. In this overview, 95.9% of the reported cases were diagnosed through NBS, highlighting its central role in case detection. Higher numbers of reported IBDD cases have come from countries where the condition is included in national or regional NBS panels, particularly in East Asia [14, 15]. This may reflect broader screening coverage and reporting practices, as well as possible population-specific patterns. Prior to the implementation of extended NBS using tandem mass spectrometry, IBDD was rarely identified and typically diagnosed in symptomatic individuals or through family-based testing.

Despite its diagnostic utility, the continued inclusion of IBDD in NBS panels remains controversial. This stems from its frequently asymptomatic presentation and the unclear long-term clinical significance of most diagnosed cases [11, 12]. Concerns have been raised about the risk of overdiagnosis, psychological burden for families, and the lack of proven benefit from early identification in individuals who remain clinically unaffected [23, 24]. While NBS has enabled the detection of rare and potentially severe phenotypes, screening programs should not be viewed primarily as tools for expanding knowledge [26]. Instead, further insights into the metabolic and clinical implications of *ACAD8* variants should be sought through targeted laboratory studies, including biochemical characterization and possibly imaging approaches such as liver ultrasound in selected cases. Clarifying the functional impact of specific variants and identifying individuals at risk for complications will be essential for directing future screening policy and follow-up strategies.

## Conclusion

This overview highlights the complex and often inconclusive nature of isobutyryl-CoA dehydrogenase deficiency (IBDD). While most individuals identified through newborn screening (NBS) remained asymptomatic, a small subset presented with variable clinical features. Although NBS has been instrumental in mapping the spectrum of IBDD, the lack of clear genotype-phenotype correlations, standardized biochemical thresholds, and long-term outcome data leaves its clinical significance uncertain. While IBDD often remains a benign metabolic trait and unnecessary medicalization of healthy individuals is of concern, this renders its inclusion to NBS panels questionable. Until more robust, long-term evidence emerges, a cautious approach to both diagnosis and screening policy remains warranted. This may include monitoring for potential hepatic alterations using abdominal ultrasound.

## Supplementary Information

Below is the link to the electronic supplementary material.

Supplementary Material 1: List of publications used specifically for patient data extraction

## Supplementary Information

Below is the link to the electronic supplementary material.

Supplementary Material 2: PRISMA 2020 checklist [33]

## Data Availability

The datasets used and/or analyzed during the current study are available from the corresponding author on reasonable request.

## Abbreviations

CoA: Coenzyme A
IBDD: Isobutyryl-coenzyme A (CoA) dehydrogenase deficiency
IBD: Isobutyryl-coenzyme A (CoA) dehydrogenase
NBS: Newborn screening
SCAD: Short-chain acyl-coenzyme A (CoA) dehydrogenase
SCADD: Short-chain acyl-coenzyme A (CoA) dehydrogenase deficiency
