# Supplementary material for: Isobutyryl-coenzyme a dehydrogenase deficiency: disease, or non-disease?
Source: Orphanet J Rare Dis. 2026 Jan 29;21:35. doi: 10.1186/s13023-026-04207-7 (PMC12857112; doi:10.1186/s13023-026-04207-7)
Supplement: Supplementary file 1 — Supplementary Material 1: List of publications used specifically for patient data extraction [file 13023_2026_4207_MOESM1_ESM.docx]

**Annex 1**

1. Afzal RM, Lund AM, Skovby F. The impact of consanguinity on the frequency of inborn errors of metabolism. Mol Genet Metab Rep. 2018;15:6–10. https://doi.org/10.1016/j.ymgmr.2017.11.004
2. Deng K, Zhu J, Yu E, Xiang L, Yuan X, Yao Y, et al. Incidence of inborn errors of metabolism detected by tandem mass spectrometry in China: A census of over seven million newborns between 2016 and 2017. J Med Screen. 2021;28(3):223–9. https://doi.org/10.1177/0969141320973690
3. Eleftheriadou M, Medici-van den Herik E, Stuurman K, van Bever Y, Hellebrekers DMEI, van Slegtenhorst M, et al. Isobutyryl-CoA dehydrogenase deficiency associated with autism in a girl without an alternative genetic diagnosis by trio whole exome sequencing: A case report. Mol Genet Genomic Med. 2021;9(2). https://doi.org/10.1002/mgg3.1595
4. Feng J, Yang C, Zhu L, Zhang Y, Zhao X, Chen C, et al. Phenotype, genotype and long-term prognosis of 40 Chinese patients with isobutyryl-CoA dehydrogenase deficiency and a review of variant spectra in ACAD8. Orphanet J Rare Dis. 2021;16(1). https://doi.org/10.1186/s13023-021-02018-6
5. Gallant NM, Leydiker K, Tang H, Feuchtbaum L, Lorey F, Puckett R, et al. Biochemical, molecular, and clinical characteristics of children with short chain acyl-CoA dehydrogenase deficiency detected by newborn screening in California. Mol Genet Metab. 2012;106(1):55–61. https://doi.org/10.1016/j.ymgme.2012.02.007
6. Hao L, Liang L, Gao X, Zhan X, Ji W, Chen T, et al. Screening of 1.17 million newborns for inborn errors of metabolism using tandem mass spectrometry in Shanghai, China: A 19-year report. Mol Genet Metab. 2024;141(1). https://doi.org/10.1016/j.ymgme.2023.108098
7. Koeberl DD, Young SP, Gregersen N, Vockley J, Smith WE, Benjamin DK, et al. Rare disorders of metabolism with elevated butyryl- and isobutyryl-carnitine detected by tandem mass spectrometry newborn screening. Pediatr Res. 2003;54(2):219–23. https://doi.org/10.1203/01.PDR.0000074972.36356.89
8. Landau YE, Waisbren SE, Chan LMA, Levy HL. Long-term outcome of expanded newborn screening at Boston children’s hospital: benefits and challenges in defining true disease. J Inherit Metab Dis. 2017;40(2):209–18. https://doi.org/10.1007/s10545-016-0004-4
9. Lee H, Lim J, Shin JE, Eun HS, Park MS, Park KI, et al. Implementation of a targeted next-generation sequencing panel for constitutional newborn screening in high-risk neonates. Yonsei Med J. 2019;60(11):1061–6. https://doi.org/10.3349/ymj.2019.60.11.1061
10. Lim JS, Tan ES, John CM, Poh S, Yeo SJ, Ang JSM, et al. Inborn error of metabolism (IEM) screening in Singapore by electrospray ionization-tandem mass spectrometry (ESI/MS/MS): An 8-year journey from pilot to current program. Mol Genet Metab. 2014;113(1):53–61. https://doi.org/10.1016/j.ymgme.2014.07.018
11. Lin Y, Peng W, Jiang M, Lin C, Lin W, Zheng Z, et al. Clinical, biochemical and genetic analysis of Chinese patients with isobutyryl-CoA dehydrogenase deficiency. Clin Chim Acta. 2018;487:133–8. https://doi.org/10.1016/j.cca.2018.09.033
12. Lin Y, Zheng Q, Zheng T, Zheng Z, Lin W, Fu Q. Expanded newborn screening for inherited metabolic disorders and genetic characteristics in a southern Chinese population. Clin Chim Acta. 2019;494:106–11. https://doi.org/10.1016/j.cca.2019.03.1622
13. Liu G, Liu X, Lin Y. Newborn screening for inborn errors of metabolism in a northern Chinese population. J Pediatr Endocrinol Metab. 2023;36(3):278–82. https://doi.org/10.1515/jpem-2022-0543
14. Navarrete R, Leal F, Vega AI, Morais-López A, Garcia-Silva MT, Martín-Hernández E, et al. Value of genetic analysis for confirming inborn errors of metabolism detected through the Spanish neonatal screening program. Eur J Hum Genet. 2019;27(4):556–62. https://doi.org/10.1038/s41431-018-0330-0
15. Oglesbee D, He M, Majumder N, Vockley J, Ahmad A, Angle B, et al. Development of a newborn screening follow-up algorithm for the diagnosis of isobutyryl-CoA dehydrogenase deficiency. Genet Med. 2007;9(2):108–16. https://doi.org/10.1097/GIM.0b013e31802f78d6
16. Pedersen CB, Bischoff C, Christensen E, Simonsen H, Lund AM, Young SP, et al. Variations in IBD (ACAD8) in children with elevated C4-carnitine detected by tandem mass spectrometry newborn screening. Pediatr Res. 2006;60(3):315–20. https://doi.org/10.1203/01.pdr.0000233085.72522.04
17. Pena L, Angle B, Burton B, Charrow J. Follow-up of patients with short-chain acyl-CoA dehydrogenase and isobutyryl-CoA dehydrogenase deficiencies identified through newborn screening: One center’s experience. Genet Med. 2012;14(3):342–7. https://doi.org/10.1038/gim.2011.9
18. Popek M, Walter M, Fernando M, Lindner M, Schwab KO, Sass JO. Two inborn errors of metabolism in a newborn: Glutaric aciduria type I combined with isobutyrylglycinuria. Clin Chim Acta. 2010;411(23–24):2087–91. https://doi.org/10.1016/j.cca.2010.09.006
19. Roe CR, Cederbaum SD, Roe DS, Mardach R, Galindo A, Sweetman L. Isolated isobutyryl-CoA dehydrogenase deficiency: An unrecognized defect in human valine metabolism. Mol Genet Metab. 1998;65(3):264–71.
20. Sadat R, Hall PL, Wittenauer AL, Vengoechea ED, Park K, Hagar AF, et al. Increased parental anxiety and a benign clinical course: Infants identified with short-chain acyl-CoA dehydrogenase deficiency and isobutyryl-CoA dehydrogenase deficiency through newborn screening in Georgia. Mol Genet Metab. 2020;129(1):20–5. https://doi.org/10.1016/j.ymgme.2019.11.008
21. Santra S, Macdonald A, Preece MA, Olsen RK, Andresen BS. Long-term outcome of isobutyryl-CoA dehydrogenase deficiency diagnosed following an episode of ketotic hypoglycaemia. Mol Genet Metab Rep. 2017;10:28–30. https://doi.org/10.1016/j.ymgmr.2016.11.005
22. Sass JO, Sander S, Zschocke J. Isobutyryl-CoA dehydrogenase deficiency: Isobutyrylglycinuria and ACAD8 gene mutations in two infants. J Inherit Metab Dis. 2004;27(6):741–5. https://doi.org/10.1023/B:BOLI.0000045798.12425.1b
23. Scolamiero E, Cozzolino C, Albano L, Ansalone A, Caterino M, Corbo G, et al. Targeted metabolomics in the expanded newborn screening for inborn errors of metabolism. Mol Biosyst. 2015;11(6):1525–35. https://doi.org/10.1039/c4mb00729h
24. Tummolo A, Leone P, Tolomeo M, Solito R, Mattiuzzo M, Lepri FR, et al. Combined isobutyryl-CoA and multiple acyl-CoA dehydrogenase deficiency in a boy with altered riboflavin homeostasis. JIMD Rep. 2022;63(4):276–91. https://doi.org/10.1002/jmd2.12292
25. Wang T, Ma J, Zhang Q, Gao A, Wang Q, Li H, et al. Expanded newborn screening for inborn errors of metabolism by tandem mass spectrometry in Suzhou, China: Disease spectrum, prevalence, genetic characteristics in a Chinese population. Front Genet. 2019;10:1052. https://doi.org/10.3389/fgene.2019.01052
26. Wang W, Yang J, Xue J, Mu W, Zhang X, Wu W, et al. A comprehensive multiplex PCR-based exome-sequencing assay for rapid bloodspot confirmation of inborn errors of metabolism. BMC Med Genet. 2019;20(1). https://doi.org/10.1186/s12881-018-0731-5
27. Yoo EH, Cho HJ, Ki CS, Lee SY. Isobutyryl-CoA dehydrogenase deficiency with a novel ACAD8 gene mutation detected by tandem mass spectrometry newborn screening. Clin Chem Lab Med. 2007;45(11):1495–7. https://doi.org/10.1515/CCLM.2007.317
28. Yun JW, Jo KI, Woo HI, Lee SY, Ki CS, Kim JW, et al. A novel ACAD8 mutation in asymptomatic patients with isobutyryl-CoA dehydrogenase deficiency and a review of the ACAD8 mutation spectrum. Clin Genet. 2015;87(2):196–8. https://doi.org/10.1111/cge.12350
29. Zhang Z, Sun Y, Wang YY, Ma DY, Wang X, Cheng W, et al. Retrospective analysis of isobutyryl-CoA dehydrogenase deficiency. Minerva Pediatr. 2024;76(5):645–51. https://doi.org/10.23736/S276.21.06179-6
30. Zhuang DY, Ding SX, Wang F, Yang XC, Pan XL, Bao YW, et al. Identification of six novel variants of ACAD8 in isobutyryl-CoA dehydrogenase deficiency with increased C4 carnitine using tandem mass spectrometry and NGS sequencing. Front Genet. 2022;12:791869. https://doi.org/10.3389/fgene.2021.791869
